# Dust, Mist and Clouds

**Published:** 1883-06

**Authors:** 


Dust, Mist and Clouds.—Whenever vapor condenses in the atmosphere, the condensation is always made on a solid nucleus, which is furnished by particles of dust. Without dust there would be neither mists nor clouds, and the super-saturated air would transform every object upon the earth's surface into a condenser upon which it would deposit its excess of water. Whenever the breath becomes visible in a cold atmosphere it demonstrates the impure and dirty condition of the air. The foam of the sea, meteoric matter and fires, are fertile sources of the dust and impurity.—*Aitkin, in Les Mondes.*

